# Investigating Molecular Connections of Non-alcoholic Fatty Liver Disease with Associated Pathological Conditions in West Virginia for Biomarker Analysis

**DOI:** 10.4172/2155-9899.1000523

**Published:** 2017-09-29

**Authors:** Dana L. Sharma, Hari Vishal Lakhani, Rebecca L. Klug, Brian Snoad, Rawan El-Hamdani, Joseph I. Shapiro, Komal Sodhi

**Affiliations:** 1 Department of Internal Medicine, Joan C. Edwards School of Medicine, Marshall University, Huntington, WV, USA; 2 Department of Surgery, Joan C. Edwards School of Medicine, Marshall University, Huntington, WV, USA

**Keywords:** NAFLD, Pbesity, Cardiovascular disease, Type 2 diabetes mellitus, Metabolic syndrome, Biomarkers, West Virginia

## Abstract

Non-alcoholic fatty liver disease (NAFLD) is a disease characterized by a steatosis of the liver that may progress to more serious pathological conditions including: nonalcoholic steatohepatitis (NASH), fibrosis, and cirrhosis. As the prevalence of NAFLD has increased worldwide in recent years, pathophysiology and risk factors associated with disease progression of NAFLD are at the focus of many studies. NAFLD is related to and shares common serum biomarkers with cardiovascular disease (CVD), type 2 diabetes mellitus (T2DM), obesity, and metabolic syndrome (MetS). West Virginia (WV) is a state with some of the highest rates of CVD, obesity and diabetes mellitus. As NAFLD is closely related to these diseases, it is of particular interest in WV. Currently there is no cost-effective, standardized method used clinically to detect NAFLD prior to the onset of reversible complications. At this time, the diagnosis of NAFLD is made with costly radiologic studies and invasive biopsy. These studies are only diagnostic once changes to hepatic tissue have occurred. The diagnosis of NAFLD by traditional methods may not allow for successful intervention and may not be readily available in areas with already sparse medical resources. In this literature review, we identify a list of biomarkers common among CVD, T2DM, obesity, MetS and NAFLD. From this research we propose the following biomarkers are good candidates for inclusion in a panel of biomarkers for the early detection of NAFLD: adiponectin, AST, ALT, apo-B, CK18, CPS1, CRP, FABP-1, ferritin, GGT, GRP78, HDL-C, IGF-1, IL-1β, 6, 8, 10, IRS-2PAI-1, leptin, lumican, MDA SREBP-1c and TNF-α. Creating and implementing a biomarker panel for the early detection and attenuation of NAFLD, prior to the onset of irreversible complication would provide maximum benefit and decrease the disease burden on the patients and healthcare system of WV.

## Introduction

Nonalcoholic fatty liver disease (NAFLD) is a growing international health problem. NAFLD is a broad diagnosis that includes: steatosis, nonalcoholic steatohepatitis (NASH), fibrosis, and cirrhosis [1]. Steatosis, characterized by an accumulation of hepatic triglycerides, is considered a benign condition [2]. As NAFLD progresses to a more serious disease, histopathologic changes like lobular inflammation (ballooning), hepatocellular damage, and fibrosis is seen. Approximately 30% of benign NAFLD diagnoses progress to NASH [2–4]. Furthermore, around 20% of NASH cases progress to liver cirrhosis and permanent end-organ disease [4]. Over the past two decades, studies have shown a substantial increase in the prevalence of NAFLD [5,6].

NAFLD/NASH has been associated with comorbidities such as type 2 diabetes mellitus (T2DM), obesity, metabolic syndrome (MetS) and cardiovascular disease (CVD). Among developed nations, the prevalence rate for NAFLD is approximately 20–40%, among these cases 70% of T2DM and as much as 95% are obese [7–10]. In addition, patients with T2DM have a prevalence rate of NAFLD 80% higher than controls [11,12]. Rates of NAFLD have increased in parallel with the obesity epidemic among both children and adults [13,14]. Wong et al. showed that since 2004, NASH has become the second leading etiology for patients awaiting liver transplants [15]. Also, NASH has been established to be a factor in increased CVD mortality rates [16–18]. In relation to MetS, NAFLD shares common risk factors, including central obesity, hypertension, insulin resistance, hyperglycemia and dyslipidemia [19,20]. It has been found that 17% of patients without MetS conditions had NAFLD, while 91% patients with all five MetS criteria had NAFLD [21].

Another related comorbidity associated with NAFLD/NASH is viral hepatitis. This disease is characterized by acute and chronic inflammation of the liver [22]. In our state of WV, viral hepatitis has placed an increasingly large burden on the population in recent years. In 2015, WV reported the highest incidence of acute hepatitis B infection and the second highest rate of acute hepatitis C infection in the United States. Further, since 2010 the rate of hepatitis B has increased by 213% and the rate of hepatitis C has increased by 209% in WV [23]. It has been observed that viral hepatitis contributes to the disease progression of NAFLD/NASH in affected patients, especially when in combination with obesity and insulin resistance [24]. In the general population, it has been observed that the prevalence of NAFLD in patients suffering from chronic hepatitis C infection is about 50% and ranges from 40% to 86% [25,26]. In turn, there is concern for the development of NAFLD/NASH due to viral hepatitis in WV. However, although this disease has been linked to the development of NAFLD/NASH and is of concern to the WV population, it is not being included in this study because this study focuses on diet-induced comorbidities of NAFLD/NASH.

NAFLD is of particular interest in our state of West Virginia (WV) since there is a high rate of NAFLD risk factors found among our population. Adults in WV have the highest prevalence of T2DM and rank second in obesity within the United States [27]. Due to the high prevalence of risk factors, we conjecture that the population of WV must also be at a great risk for the development of NAFLD. With this in mind, we have conducted this literature review for the purpose of creating a panel of biomarkers to correlate with CVD, T2DM, obesity, and MetS. This biomarker panel could provide a minimally invasive, cost effective method to assess patients for NAFLD/NASH. Furthermore, with this panel, patients will have the means for early detection, which provides an opportunity to diminish the overall prevalence of NAFLD/NASH. Using a panel of biomarkers allows for the assessment of a variety of metabolic pathways. Additionally, a panel could provide a mechanism to personalize treatments geared towards particular metabolic derangements defined by the panel. We have compiled a list of the most prevalent biomarkers in the literature and if applied to our population at risk would provide a way to identify, monitor, and potentially treat patients in a minimally invasive, cost-effective way.

## Cardiovascular Disease

CVD affects a great number of adult populations worldwide and leads to several life-threatening conditions including myocardial infarction (MI), congestive heart failure (CHF), coronary artery disease (CAD), stroke, peripheral vascular disease, hypertension and atrial fibrillation. Around 82.6 million people in United States have at least one form of CVD. CVD is the most common cause of death among all adults [28]. Some of the highest rates of CVD are observed in the population of WV, since there are high rates of risk factors such as diabetes and obesity [29,30]. Evidence is presented that associates NAFLD with CVD. This relationship suggests increased risk and progression to CVD in patients who have been diagnosed with NAFLD [31]. The pathogenic progression of NAFLD suggests that it exasperates the formation of vascular plaque perpetuating CVD [31].

Examination of the link between NAFLD and CVD yielded several inflammatory biomarkers common in the progression of both diseases. TNF-α has been suggested as one of the pro-inflammatory cytokines aiding in the progression of NAFLD [32]. Up-regulation of TNF-α cause’s systematic inflammation of lipids and activation of oxidative stress mechanisms involved in the development of CVDs. Furthermore, among obese and patients with insulin resistance, dysregulated cytokines, IL-8 and IL-10 contribute to the progression of NAFLD. A correlation in the levels of IL-8 and IL-10 exists among patients with CVD and NAFLD [33,34]. Several studies illuminate an association of the fibrinolytic marker, Plasminogen Activator Inhibitor 1 (PAI-1), in the progression of NAFLD and CVD [32]. Studies show that PAI-1 is partially responsible for vascular thrombotic and fibrotic events, which contribute to the macro-vascular complications of obesity, diabetes and NAFLD [35–37]. Multiple studies have closely examined the circulating levels of PAI-1 in patients with NAFLD, this data showed a correlation between elevated levels of PAI-1 and CVD [35]. Another biomarker, Sterol Regulatory Element Binding Protein-1c (SREBP-1c), has been measured at levels almost 5 times greater in patients with NAFLD than the control samples. SREBP-1c advances NAFLD by contributing to the up-regulation of fatty acid synthesis [38]. Furthermore, as hyperlipidemia is a risk factor for NAFLD, those with NAFLD have elevated levels of specific lipoproteins like apoB, compared to populations without this liver disease. The aberrant pathophysiological activity of lipoprotein metabolism aggravates the development of atherogenesis in this population [32]. From the literature, several biomarkers that patients with NAFLD and CVD share in common include: TNF-α, IL-8, IL-10, PAI-1, SREBP-1c and apoB. Finding common biomarkers highlights the interconnections between the two disease states.

## Diabetes Mellitus

Diabetes mellitus (DM) is a disease rising to epidemic proportions [39]. In 2012 alone, diabetes mortality figures were equal to those of AIDS/HIV and the World Health Organization predicts diabetes will be the seventh leading cause of death in the world by 2030 [40]. The connection between DM and NAFLD/NASH is based on the pathophysiology of insulin resistance, specifically in relation to T2DM. Insulin resistance in NALFD/NASH patients increases a patient’s risk of developing T2DM and vice versa [41].

Recent research into the relationship between NAFLD/NASH and DM has led to the discovery of a variety of biomarkers consistent between the diseases. Adiponectin is a hormone produced by adipocytes; serum levels of this hormone are reduced in insulin resistance. Normally this hormone acts to promote insulin sensitivity by fatty acid oxidation and hepatic glucose production. Adiponectin levels are decreased during states of inflammation, especially by cytokines like tumor necrosis factor-alpha (TNF-α) [42]. As insulin resistance is a major factor for T2DM and NAFLD/NASH development, adiponectin is decreased in both diseases. Additionally, adipocyte dysregulation is directly related to the development of steatohepatitis; this provides evidence to suggest that adiponectin is a good choice as a biomarker for both T2DM and NAFLD [43,44]. Another molecule, C-reactive protein (CRP) is released acutely during the-inflammatory response [43]. Consistent CRP elevation positively correlates with the development of diabetes later in life. Chronic inflammation is marked by insulin resistance and henceforth, over time this results in the development of T2DM. Higher HbA1c levels were associated with higher CRP levels, pre-diabetes and T2DM [45,46]. Reduced glucose tolerance and the presence of NAFLD/NASH were associated with increased CRP levels [43,47,48]. Additionally, TNF-α is an acute phase pro-inflammatory protein, perpetuating insulin resistance, hepatic lipogenesis, and serum triglyceride levels [43]. Increased hepatic lipogenesis and serum triglyceride levels are main causes of NAFLD/NASH [49]. Studies have also found that interleukin-1 beta (IL-1β), released during inflammatory responses, aggravates chronic disease causing acute tissue damage [50]. Elevated levels of IL-1β serve as a predictor of future T2DM development [51]. Interleukin-6 (IL-6) is a pro-and anti-inflammatory cytokine. Both IL-1β and IL-6 are found at higher levels in patients with diabetes and NAFLD/NASH than in control groups [51–54]. Interleukin-10 (IL-10) is an anti-inflammatory cytokine, these levels appear decreased in patients with T2DM and NALFD/NASH; this is fitting as both diseases involve a steady undercurrent of inflammation [52,55]. In addition, insulin receptor substrate-2 (IRS-2) expression impacts the effects of insulin in the bloodstream. DM and NAFLD/NASH have reduced levels of IRS-2 expression. Lower levels of IRS-2 expression cause decreased lipid metabolism, increased insulin resistance, and alters hepatic nutrient homeostasis [38,56–58]. Studies have also shown that alanine aminotransferase (ALT) and gamma-glutamyl transferase (GGT), enzymes produced mostly in the liver, are elevated in cases of T2DM and NAFLD/NASH [46,59,60]. Recent research also finds a direct relationship between ferritin levels, an iron storing protein, HbA1c and serum insulin levels. Elevated ferritin levels are considered indicative of future development of T2DM [61]. Since ferritin is stored in hepatic cells, liver damage causes a release of ferritin into the bloodstream. Therefore, elevated serum ferritin could be characteristic of liver necrosis [62]. Ferritin is associated with inflammation and is increased in T2DM as well as in NAFLD/NASH [63].

## Obesity

Obesity is the most common metabolic disease in the world [64]. It occurs due to a variety of lifestyle/genetic factors which cause an imbalance in the amount of energy consumed as compared to the amount of energy expended [65]. It is a chronic disease, characterized by the presence of excess adipose tissue and overproduction and secretion of inflammatory factors or coagulation molecules from adipocytes [65,66]. Of particular interest is the health risks associated with obesity. These include the development of insulin resistance and T2DM, hypertension, CVD, hypertriglyceridemia, and dyslipidemia [64–67]. Notably, there is also a significant correlation between obesity and NAFLD. The reported prevalence of NAFLD is around 80% in obese patients [68,69].

Common biomarkers between obesity and NAFLD/NASH have been recently identified. Studies have shown that lowered levels of plasma adiponectin are closely associated with obesity and the development of NASH [42,70]. Furthermore, increased serum levels of alanine aminotransferase (ALT) and cytokeratin-18 (CK18) fragments, two markers associated with liver damage as well as NAFLD/NASH, are applicable to predict NASH progression in morbidly obese patients [71–75]. Low serum concentrations of interleukin-6 (IL-6), a cytokine involved in the immune response and insulin-like growth factor 1 (IGF-1), a peptide involved in carbohydrate metabolism, are correlated with progression to NASH in obese patients [76,77]. Additionally, carbamoyl phosphate synthetase I (CPS1), glucose regulated protein 78 (GRP78), and fatty acid binding protein-1 (FABP-1) are all under-expressed in obese NASH patients as compared to obese patients without NASH [78,79]. Lumican, a keratin sulfate proteoglycan, is overexpressed in obese patients NASH, thus indicating its potential as a biomarker for NASH in obese patients [79,80]. Levels of C-reactive protein (CRP), a peptide involved in the inflammatory response, has a direct relationship to the degree of steatosis in obese patients, indicating its usefulness as a biomarker [81]. Additionally, although they are not serum biomarkers, abnormal glucose metabolism and systemic hypertension have also been identified as effective predictors of the development of NASH in obese patients [75].

## Metabolic Syndrome

Metabolic irregularities: dyslipidemia, hyperglycemia, insulin resistance, hypertension and obesity are components of MetS; this is a progressive condition that encompasses a variety of pathologies [29]. With a prevalence of 34%, MetS is one of the most common diseases in the United States; however, the mechanisms of MetS pathogenesis are still unclear and require further research [82,83]. A strong correlation between MetS and NAFLD/NASH has been established and MetS has already been identified as a strong predictor of the development of NAFLD [84–87]. Based on this correlation, research has been conducted concerning MetS and NAFLD/NASH in an attempt to gain more knowledge on the relationship between these two diseases.

Previous research defined a relationship between MetS and NAFLD/NASH, and identified a variety of biomarkers that may detect NAFLD/NASH. One study conducted found a positive correlation between the presence of MetS, increased levels of certain biomarkers, steatohepatitis and NAFLD. These markers include high-sensitivity C-reactive protein (hs-CRP), malondialdehyde (MDA), AST, ALT, circulating triglyceride levels and inverse high-density cholesterol (HDL-C) [88]. Other biomarkers identified in the literature correlating MetS and NAFLD/NASH include: increased levels of IL-6, leptin, and TNF-α as well as decreased levels of adiponectin [89–91]. These markers have also been identified with similar trends that correlate with disease progression of MetS [29,92–96]. These trends in biomarkers between these two diseases outlines the value of noninvasive tests to identify disease progression MetS leading to NAFLD/NASH.

## Discussion

In this paper we examined biomarkers, from existing literature, that could be considered for a panel of biomarkers for the early detection of NAFLD. The biomarkers that we examined, exhibit overlap between NAFLD and other metabolic diseases that are known risk factors for NAFLD: CVD, T2DM, obesity and MetS. As WV is home to a population with the highest rates of these risk factors, NAFLD is of great concern to the health of our population. Unfortunately, NAFLD cannot be diagnosed with current methods, prior to the onset of permanent tissue damage.

**Figure 1:**
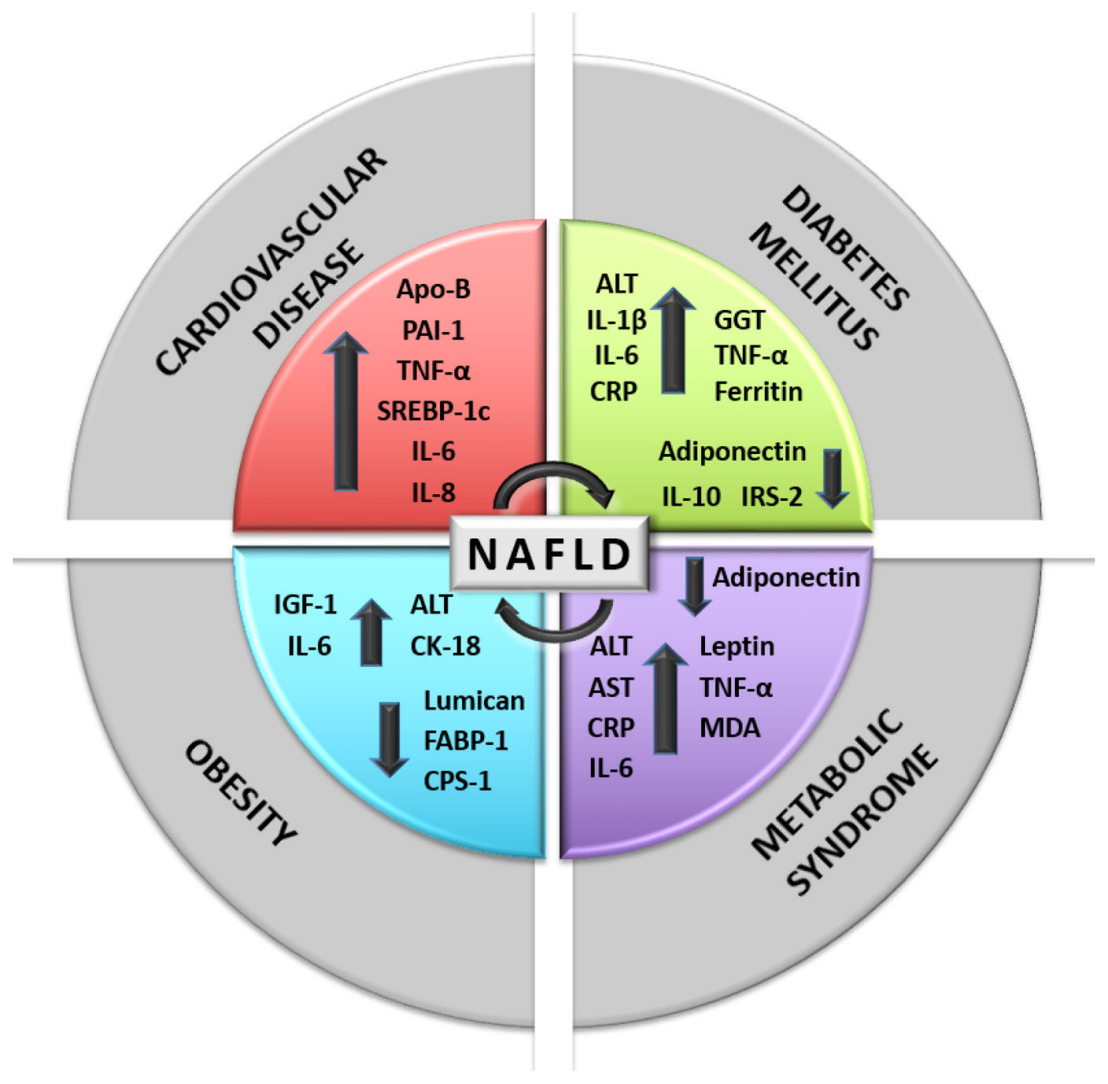
Common Biomarkers of NAFLD, CVD, T2DM, MetS and Obesity. A schematic overview displaying the interactions of cytokines, inflammatory markers and adipokines resulting from CVD, DM, obesity and MetS contributing to the development of NAFLD.

Developing a blood test that promotes an early diagnosis and disease monitoring of NAFLD would be immensely valuable to the health of the populations with high rates of obesity and other metabolic diseases. As NAFLD, CVD, Obesity, T2DM and MetS are multifactorial, having a panel of several biomarkers is a valuable tool to monitor the onset and progression of this disease. Further studies are needed to delineate the best biomarkers for early stage diagnosis of NAFLD among our population. The biomarkers examined include hormones, inflammatory cytokines and other molecular active proteins: adiponectin, AST, ALT, apo-B, CK18, CPS1, CRP, FABP-1, ferritin, GGT, GRP78, HDL-C, IGF-1, IL-1β, 6, 8, 10, IRS-2PAI-1, leptin, lumican, MDA SREBP-1c and TNF-α (Figure 1). Clinical application of such a panel would provide ample time for clinicians to properly treat their patients and provide the potential for disease prevention. Access to this blood test for early diagnosis of NAFLD would be of great benefit to areas with decreased access to healthcare resources and it would be less expensive than current methods.

## Conclusion

NAFLD is a condition closely associated with CVD, T2DM, obesity and MetS with an increasing prevalence in populations worldwide, especially in WV. High rates of related conditions such as T2DM and obesity have lead NAFLD to become a rising concern across the state. This panel of biomarkers demonstrates the progression from associated diseases to NAFLD and may provide a way to detect this disease prior to the onset of irreversible complications. This would provide time for interventions such as: lifestyle modifications, medications and further screening and in turn, decrease the disease burden on the patients, healthcare system and resources available in WV.
